# Iatrogenic topiramate poisoning in an ICU patient: Focus on topiramate peak time prolongation 

**DOI:** 10.5414/CP204067

**Published:** 2021-10-08

**Authors:** Vera M.G. Hoebregts, Norbert Foudraine, Paddy K.C. Janssen, Jos L.M.L. le Noble

**Affiliations:** 1 Department of Clinical Pharmacy, Pharmacology and Toxicology, Zuyderland Medical Center, Sittard-Geleen,; 2 Department of Intensive Care,; 3 Department of Hospital Pharmacy, VieCuri Medical Center Venlo,; 4 Department of Clinical Pharmacy and Toxicology, Maastricht University Medical Center, and; 5 Department of Pharmacology and Toxicology, Maastricht, The Netherlands

**Keywords:** toxicokinetics, topiramate, poisoning, ICU, therapeutic drug monitoring

## Abstract

A 35-year-old man with generalized insults was admitted to the intensive care unit because of third-line treatment of persistent epileptic insults with antiepileptic drug therapy. Topiramate was added on top of his outpatient regimen in combination with intravenous antiepileptic drugs. Miscommunication and inappropriate topiramate dosing (2,500 mg twice) resulted in an acute topiramate intoxication. Toxicokinetic assessment showed toxic serum topiramate concentration of 55 mg/L and a dose-dependent shift of peak time t_max_. According to our modulations, t_max_ follows Y = 0.0009X + 2.65, where X is the topiramate dose. Our results have important implications for effectiveness of gut decontamination modalities.

**What is known about this subject**

- Topiramate intoxications can lead to coma, confusion, somnolence, and seizures. Gut decontamination should be applied immediately before t_max_ is reached because increasing time between topiramate intake and decontamination will decrease efficacy to decontaminate. However, limited data exist on pharmacokinetics during topiramate overdose, and current consensus guidelines of gut decontamination do not include shifts of t_max_ during topiramate intoxication.

**What this study adds**

Our case is the first case to describe the following:

- t_max_ in topiramate intoxication may considerably increase in a dose-dependent manner following the equation Y = 0.0009X + 2.65 (in which Y is t_max_ (h), where X is the topiramate dose (mg)).
- Extended t_max_ can cause symptoms later than expected.
- For clinicians, it is important to note that present guidelines for gut decontamination for acute poisoning during topiramate overdose do not include shifts with an increase in t_max_. Beneficial effects of gastrointestinal decontamination persist longer in patients with topiramate intoxication due to lengthened t_max_.

## Introduction 

Topiramate is a broad-spectrum anticonvulsant drug (AED). Acute poisoning with topiramate primarily involves the central nervous system [1]. Antidotes are lacking in case of poisoning [1, 2]. General measures include support of vital functions [1, 2]. Charcoal gastrointestinal decontamination can yield treatment benefits [3], but its effectiveness beyond the generally accepted time window of 1 – 3 hours remains to be elucidated. 

We report a case of an intensive care unit (ICU) patient with topiramate toxicity from an unintended overdose due to prescribing errors. We assessed the effectiveness of gastrointestinal decontamination related to topiramate peak time based on data from our patient and those previously published. 

## Case report 

A 35-year-old Caucasian male weighing 124 kg (body mass index: 34 kg/m^2^) with a history of epilepsy was admitted to the ICU with generalized seizures. On arrival, the patient was unresponsive, and his body temperature was 35.9 °C. His blood pressure was 194/119 mmHg, pulse rate was 100/minute, and respiratory rate was 18/minute. The clinical assessment of disease severity according to the APACHE IV score was 92. Renal and liver tests were normal. Endotracheal intubation was performed. Lacosamide and levetiracetam were administered intravenously twice daily (50 mg and 1,500 mg, respectively). On day 4, due to increased antiepileptic activity, 250 mg topiramate twice daily was prescribed. 

Due to miscommunication, a dose of 2,500 mg topiramate was administered by a nasogastric tube. The next day, the prescription error was discovered but a toxicity syndrome was already suspected. Prompt gastrointestinal decontamination, including active charcoal, was initiated within 12 hours from administration. A laxative (sodium thiosulphate) was administered every 4 hours to prevent drug absorption. No seizures were recorded on the EEG. The patient recovered fully and was discharged on day 6. 

## Topiramate plasma concentrations and toxicokinetics 

The data set of our patient consisted of five serial pharmacokinetic concentration time profiles of topiramate representing the elimination phase. Topiramate plasma concentrations were determined in left over samples. The peak plasma concentration of topiramate was 55 µg/mL. Table 1 shows the pharmacokinetic parameters of topiramate of our patient, the population average, and a case reported by Brandt et al. [2]. After the t_max_, first-order kinetics could be used to fit topiramate clearance. Similar simulations were done for standard oral dosing of 100 mg topiramate. Finally, we used 10 serial pharmacokinetic concentration time profiles representing the elimination phase (first-order kinetics) from a patient with a topiramate intoxication published by Brandt et al. [2]. 

**Figure 1:**
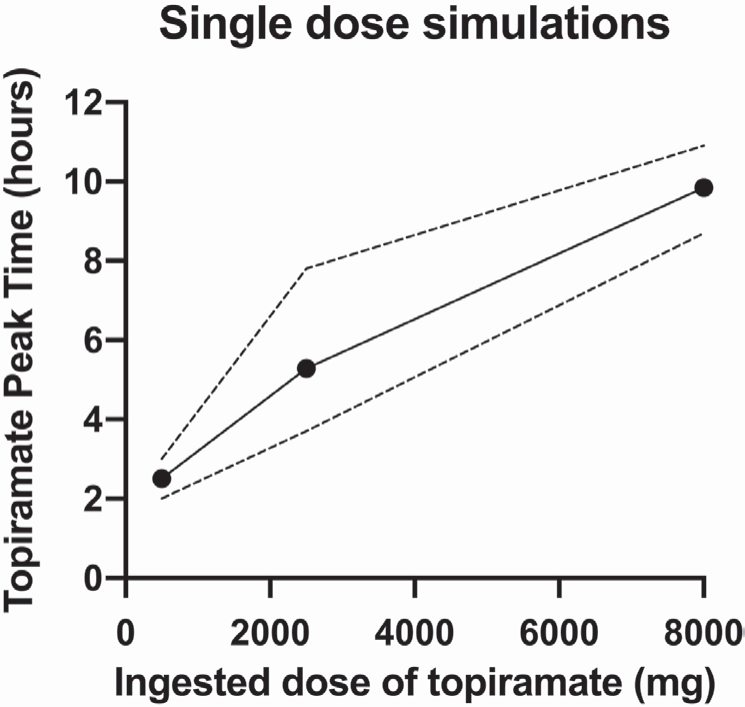
The best-fit linear regression line showing a linear relationship between ingested dose and topiramate peak time, represented graphically with the ingested dose on the X-axis and the peak time on the Y-axis (Y = 0.0009X + 2.65). The dotted line represents the maximum ranges to either side. The left, middle, and right data points represent patients 1, 2, and 3, respectively, presented in Table 1. Data points represent means of experimental data.

Figure 1 presents the topiramate peak time and ranges plotted against the topiramate dosage based on the data set from Table 1. The best-fit linear regression line showed a linear relationship between ingested dose and topiramate peak time, represented graphically with the ingested dose on the X-axis and the peak time on the Y-axis (Y = 0.0009X + 2.65). 

The oral topiramate doses used for the simulations were 250 mg as standard dose, 2,500 mg for the present patient, and 8,000 mg for the dose obtained from available data published by Brandt et al. [2]. t_max_ (h) is presented as mean values with ranges. 

## Discussion 

The topiramate dose administered in our patient was 10 times the prescribed dose, causing acute toxicity. Our pharmacokinetic modeling demonstrated a prolongation of t_max_ during topiramate toxicity, which occurred linearly in a dose-dependent manner (Figure 1) for up to 10 hours. Delayed gastric emptying as a result of narcotic administration or diminished splanchnic blood flow may have contributed to the delay in t_max_ that we found. 

In case of absence of a topiramate toxicity syndrome in the first hours of ingestion, it could be falsely interpreted as favorable and may lead to delayed supportive treatment. Our case aligns with experimental data showing that peak times following a single dose appear earlier when the patient is receiving repeated consistent doses over time, but that it is longer when the patient receives a single new dose [4]. Furthermore, topiramate elimination is capacity-limited [2]. Other agents may have changed the absorption rate in the gastrointestinal tract [5], and obesity in our patient might have affected liver function and topiramate kinetics [2]. 

## Conclusion 

With our newly developed equation (Y = 0.0009X + 2.65), clinicians can easily determine the expected peak time of topiramate. Moreover, we have shown that patients may still benefit from gastrointestinal decontamination up to 10 hours following ingestion of toxic doses. 

## Acknowledgment 

We would like to thank Hai Holthuysen, a laboratory technician at the VieCuri Medical Center for the determination of topiramate. 

## Funding 

The authors declare that no external funding was received for the conduct of this study and/or the preparation of this manuscript. 

## Conflict of interest 

Authors declare no conflict of interest. Authors declare that they have no commercial or proprietary interest in any drug, device, or equipment mentioned in the article. 

**Table 1.** Pharmacokinetic modeling using MWPharm 3.58 of three different oral doses of topiramate: 100, 2,500, and 8,000 mg.

	General population	Present case report	Patient by Brandt et al. [2]
Ingested dose (mg)	100	2,500	800
t_max_ (h), ranges	2.5 (2 – 3)	5.28 (3.6 – 7.8)	9.84 (8.7 – 10.9)
T_1/2_ (h)	± 21	16.48	23.26
V_d_ (L/kg)	0.55 – 0.80	0.69	0.46
Total body clearance (L/h)	1.7 – 1.8	2.39	1.04

The pharmacokinetic parameters were assessed in: 1. an adult healthy subject using published clinical PK data from the Summary of Product Characteristics of Topamax (Janssen-Cilag B.V., Breda, The Netherlands) [6]; 2. the present patient using experimental data of 5 measurements; 3. a patient with a topiramate intoxication described by Brandt et al. [2] using experimental data of 10 measurements. t_max_ (h) values are presented as mean and ranges. Drug dosing and calculation of the volume of distribution (L/kg) were based on lean body mass.

## References

[1] Lynch MJ Pizon AF Siam MG Krasowski MD Clinical effects and toxicokinetic evaluation following massive topiramate ingestion. J Med Toxicol. 2010; 6: 135–138. 20376593 10.1007/s13181-010-0065-y PMC2916051

[2] Brandt C Elsner H Füratsch N Hoppe M Nieder E Rambeck B Ebner A May TW Topiramate overdose: a case report of a patient with extremely high topiramate serum concentrations and nonconvulsive status epilepticus. Epilepsia. 2010; 51: 1090–1093. 19889015 10.1111/j.1528-1167.2009.02395.x

[3] Thanacoody R Caravati EM Troutman B Höjer J Benson B Hoppu K Erdman A Bedry R Mégarbane B Position paper update: whole bowel irrigation for gastrointestinal decontamination of overdose patients. Clin Toxicol (Phila). 2015; 53: 5–12. 25511637 10.3109/15563650.2014.989326

[4] Greenberg HE England MJ Hellriegel ET Bjornsson TD Time of peak drug concentration after a single dose and a dose at steady state. J Clin Pharmacol. 1997; 37: 480–485. 9208354 10.1002/j.1552-4604.1997.tb04325.x

[5] Yamaguchi J Kinoshita K Noda A Furukawa M Sakurai A Delayed increase in serum acetaminophen concentration after ingestion of a combination medications: a case report. J Int Med Res. 2018; 46: 3435–3439. 29882461 10.1177/0300060518777860 PMC6134647

[6] Janssen-Cilag BV Summary of Product Characteristics. Topamax 25, 50, 100, 200 mg. 2010. URL: https://www.geneesmiddeleninformatiebank.nl/smpc/h24167_smpc.pdf.Date of access: May 22, 2021.

